# What works for whom in pharmacist-led smoking cessation support: realist review

**DOI:** 10.1186/s12916-016-0749-5

**Published:** 2016-12-16

**Authors:** Trisha Greenhalgh, Fraser Macfarlane, Liz Steed, Robert Walton

**Affiliations:** 1 Nuffield Department of Primary Care Health Sciences, University of Oxford, Oxford, UK; 2 Asthma UK Centre for Applied Research, Centre for Primary Care and Public Health, Barts and The LondonSchool of Medicine and Dentistry, Queen Mary University of London, London, UK

## Abstract

**Background:**

New models of primary care are needed to address funding and staffing pressures. We addressed the research question “what works for whom in what circumstances in relation to the role of community pharmacies in providing lifestyle interventions to support smoking cessation?”

**Methods:**

This is a realist review conducted according to RAMESES standards. We began with a sample of 103 papers included in a quantitative review of community pharmacy intervention trials identified through systematic searching of seven databases. We supplemented this with additional papers: studies that had been excluded from the quantitative review but which provided rigorous and relevant additional data for realist theorising; citation chaining (pursuing reference lists and Google Scholar forward tracking of key papers); the ‘search similar citations’ function on PubMed. After mapping what research questions had been addressed by these studies and how, we undertook a realist analysis to identify and refine candidate theories about context-mechanism-outcome configurations.

**Results:**

Our final sample consisted of 66 papers describing 74 studies (12 systematic reviews, 6 narrative reviews, 18 RCTs, 1 process detail of a RCT, 1 cost-effectiveness study, 12 evaluations of training, 10 surveys, 8 qualitative studies, 2 case studies, 2 business models, 1 development of complex intervention). Most studies had been undertaken in the field of pharmacy practice (pharmacists studying what pharmacists do) and demonstrated the success of pharmacist training in improving confidence, knowledge and (in many but not all studies) patient outcomes. Whilst a few empirical studies had applied psychological theories to account for behaviour change in pharmacists or people attempting to quit, we found no studies that had either developed or tested specific theoretical models to explore how pharmacists’ behaviour may be affected by organisational context. Because of the nature of the empirical data, only a provisional realist analysis was possible, consisting of five mechanisms (pharmacist identity, pharmacist capability, pharmacist motivation and clinician confidence and public trust). We offer hypotheses about how these mechanisms might play out differently in different contexts to account for the success, failure or partial success of pharmacy-based smoking cessation efforts.

**Conclusion:**

Smoking cessation support from community pharmacists and their staff has been extensively studied, but few policy-relevant conclusions are possible. We recommend that further research should avoid duplicating existing literature on individual behaviour change; seek to study the organisational and system context and how this may shape, enable and constrain pharmacists’ extended role; and develop and test theory.

## Background

New models of primary care are urgently needed to address funding and staffing pressures. One such model is an extended role for the community pharmacist. In the UK, for example, the contract for community pharmacists was revised in 2005 to embrace three levels of service: essential (basic dispensing and medicines advice), advanced (e.g. extended professional services such as smoking cessation support and medicines use review), and enhanced (locally commissioned services to address local priorities) [1]. As in many other countries, the community pharmacy is seen as an accessible and potentially cost-effective venue for providing a range of public health services and targeting particular needs at a time when general practice is under unprecedented strain [2]. Yet, uptake of the extended role of the pharmacist has been lower than expected both in the UK and elsewhere [2, 3]. Smoking cessation is a priority area for health promotion and one in which pharmacists showed early interest [4].

The STOP programme is a mixed-methods research study that aims to answer the over-arching research question “Will a personalised smoking cessation intervention delivered in community pharmacies (and including an education programme to develop workers’ engagement and consultation skills) improve engagement in the NHS Stop Smoking service and quit rate in smokers?” [5].

The other elements of the STOP programme are (1) a systematic review of behavioural interventions in community pharmacies, beginning with a quantitative review of (mostly) randomised controlled trials (RCTs) but including other controlled designs, according to Cochrane Effective Practice and Organisation of Care (EPOC) criteria [6]; (2) qualitative observational studies of pharmacist–patient consultations relating to smoking cessation; (3) development and piloting of an educational intervention for community pharmacy workers; and (4) a pragmatic cluster randomised trial (1200 patients, 60 pharmacies) to evaluate the effectiveness of the educational intervention in both promoting uptake of the NHS Pharmacy Smoking Cessation Service and increasing quit rates. The trial will also collect data on costs and adverse events.

This study, part of the qualitative and mixed-methods component of our background literature review, aimed to identify and analyse theoretical and empirical data on the context and process of pharmacist-led smoking cessation support programmes.

### Aims

To identify what works for whom in what circumstances in relation to smoking cessation support delivered in community pharmacies.

### Objectives

#### Strategic objectives

To explain successes, failures and partial successes in published empirical studies of behavioural interventions delivered by pharmacists, and in particular to explain heterogeneity in the findings of RCTs.

#### Operational objectives

1. Identify studies of pharmacy-based smoking cessation interventions.
2. Identify additional relevant publications that would contribute to theory building about what works for whom in what circumstances.
3. Gain familiarity with the dataset by close reading.
4. Produce a descriptive summary of the data to summarise what kinds of research question have been asked, how these questions have been addressed and what the key findings are to date.
5. Develop a realist analysis consisting of candidate theories linking context, mechanism and outcome.
6. Undertake systematic data extraction to test and refine these candidate theories.
7. Summarise the middle-range theories for which there is strong empirical evidence of what works for whom in what circumstances.
8. Clarify gaps in the knowledge base and make recommendations for further research.

## Methods

### Study design

Descriptive synthesis and realist review conducted according to RAMESES standards.

### Realist review

The principles of realist review have been described in more detail elsewhere [7]; we summarise them briefly here.

The realist research question is often summarised as “What works for whom in what circumstances, how and why?” [7]. Realist inquiry considers the interaction between context (the organisational and system setting in which complex interventions are delivered), mechanism (how the intervention works) and outcome. Complex interventions rarely have a constant effect size. They are usually more effective in some circumstances than others. Intervention X (e.g. a programme introduced by policymakers who seek to create a particular outcome) alters context (for example, by making new resources available), which then triggers mechanism(s) (“*…underlying entities, processes, or* [social] *structures which operate in particular contexts to generate outcomes of interest*” [8]), which produce both intended and unintended outcomes.

Realist inquiry seeks to unpack the context–mechanism–outcome relationship, thereby explaining examples of success, failure and various eventualities in between. Theoretical explanations of this kind are referred to as “middle-range theories” (i.e. ones which involve a certain amount of abstraction but which are close enough to observed data to be incorporated in propositions that permit empirical testing). Examples of middle-range theory are given later in the paper.

An international Delphi panel published the RAMESES quality standards and publication guidance for realist review in 2013 [9]. This review was undertaken with reference to those standards.

### Research questions

As is usually the case in realist review, our over-arching research question “what works for whom in what circumstances in relation to the role of community pharmacies in providing lifestyle interventions to support smoking cessation?” was refined to a number of more specific questions as the review unfolded. These comprised five descriptive questions and one higher-order (realist) question. Respectively, these were:

1. What is the impact on pharmacists of training them in smoking cessation support?
2. What is the perspective of patients and the public on pharmacy-based smoking cessation support?
3. What do pharmacists perceive are the barriers to delivering smoking cessation support?
4. What do pharmacists believe would help them to deliver smoking cessation support?
5. How do organisational and system factors influence uptake and delivery of smoking cessation counselling by pharmacists?
6. How do the key mechanisms of pharmacist identity, pharmacist capability, pharmacist motivation, clinician confidence and public confidence interact with contextual influences and with one another to explain the successes, failures and partial successes of pharmacy-based smoking cessation schemes?

### Sample

**Fig. 1:**
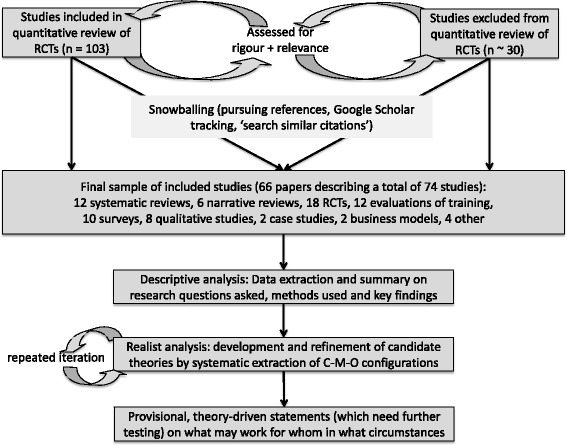
Study flowchart

Data collection occurred from January to June 2016. Our sampling strategy is summarised in the flowchart (Fig. 1).

We began with the sample of studies included in the STOP quantitative review [10]. These had been identified previously through systematic searching of seven databases (Cochrane Central Register of Controlled Trials, Health Technology Assessment Database, NHS Economic Evaluation Database, Medline including in-process citations, EMBASE, PsycINFO, EPOC). These studies consisted mostly of RCTs of community pharmacy-based behavioural interventions, in relation to both smoking cessation and a range of other behaviours (e.g. weight management, sexual health, asthma). They also included linked (‘sister’) publications, that is, papers by the same authors, describing further analysis or commentary on the same original dataset and/or additional empirical studies, qualitative evaluations, critiques or commentaries by other authors.

For the purposes of this realist review, we were interested less in quantitative findings of intervention studies (e.g. effect sizes) per se than in the nuanced explanations of why a particular intervention had been more or less successful at influencing the target behaviour. Therefore, not all the studies in this sub-sample were relevant. One of us (TG) reviewed the full-text papers and made a judgement as to whether the paper included sufficient descriptive detail and/or theoretical discussion to contribute to the development and/or testing of theory-driven explanations about why and how a community pharmacy delivered smoking cessation intervention might work. Studies lacking descriptive detail were not considered further. A 10% sub-sample of included and excluded papers was checked by FM; differences were minimal.

We supplemented this initial sample with relevant additional papers from three sources. First, we also considered studies that had been excluded from the EPOC review of quantitative findings but which provided rigorous and relevant additional data for realist theorising. Aspects of a study may be relevant for theory building even when the study’s main research question is marginal or when some aspects of the study (e.g. outcome measures) did not meet the review’s inclusion criteria. In particular, some studies had been rejected from the EPOC review because they did not have a clinically validated measure of smoking cessation but nevertheless could contribute key contextual detail to this theory-focused review.

Second, taking account of the efficiency and effectiveness of ‘fuzzy’ search methods in identifying key papers in reviews of complex and heterogeneous topic areas [11], we undertook citation chaining (pursuing reference lists and Google Scholar forward tracking) of all papers that we judged to be centrally relevant to our realist question. The reference lists of these papers identified studies that had been published prior to them, and Google Scholar identified studies published subsequently that cited them.

Third, and again reflecting the value of ‘fuzzy’ search methods, we used the ‘search similar citations’ function on PubMed. We entered the title of each key paper in turn, and considered the titles of studies offered by the software as ‘similar’ to the one entered.

In each of these three steps, many papers could be excluded as irrelevant on title and abstract alone, but where necessary we obtained the full text of the paper to consider whether it included sufficient detailed description on a rigorous and relevant aspect of the study and/or sufficient theoretical discussion to add to our emerging synthesis – and in particular whether it might contribute to the development and refinement of programme theories. Judgements were made by TG and a 10% sample checked by FM with no substantial discrepancies.

### Descriptive analysis

In order to gain familiarity with the data, all full-text papers deemed relevant were initially read and re-read by FM and TG, and a preliminary thematic analysis undertaken using a combination of paper annotations and an Excel spreadsheet to aid data management. In this phase, we sought to gain an overview of how researchers had studied the provision of smoking cessation support by pharmacists, to identify examples of highly successful programmes and also unsuccessful ones, and to highlight explanations (e.g. from the discussion sections of the empirical studies) that could serve as candidate theories to be tested further in the next phase of the study.

We used an Endnote database to classify each publication in each of four categories: study design, academic discipline, country and methodological rating.

- ‘Study design’ included primary designs (e.g. RCT, qualitative study) and secondary designs (e.g. systematic review of RCTs, systematic review of qualitative studies).
- ‘Academic discipline’ referred to the broad research tradition in which the study was located (assessed by judging the academic department of the lead author, the journal in which it was published and the literature it cited). Examples of academic disciplines were public health (an interdisciplinary field of study focused on the health of communities or sub-populations), pharmacy practice (the study by pharmacists of what pharmacists do), and health economics (the study of the costs and cost-effectiveness of health interventions).
- ‘Country’ referred to the country in which a primary empirical study had been undertaken; for secondary research, it was the country of the lead author.
- ‘Methodological rating’ was an estimate (on a 3-point scale) of the methodological quality of the study. We gave three stars to studies that had no major flaws within their genre and were adequately powered to provide a definitive answer to the authors’ research question; two stars to studies that were too small to provide definitive answers but were otherwise methodologically robust, and one star to studies that had significant flaws (most commonly small studies in a parochial setting whose findings could not be generalised with any confidence).

The methodological rating for each individual study was used as an interim metric to contribute to a modified ‘strength of evidence’ score based on the World Health Organization Health Evidence Network criteria [12]:

- Strong direct evidence: consistent findings in two or more empirical studies of appropriate design and high scientific quality (3 stars in our rating) relating directly to the provision of smoking cessation support by community pharmacists or pharmacy workers overseen by pharmacists.
- Strong indirect evidence: consistent findings in two or more empirical studies of appropriate design and high scientific quality, relating to the provision of some other behavioural intervention by community pharmacists or pharmacy workers.
- Moderate direct (or indirect) evidence: consistent findings in two or more empirical studies of less appropriate design and/or of acceptable scientific quality (two stars in our rating) relating directly to the provision of smoking cessation support (or indirectly to the provision of some other behavioural intervention) by community pharmacists or pharmacy workers overseen by pharmacists.
- Limited evidence: only one study of appropriate design and acceptable quality (two stars) available, or inconsistent findings in several studies.
- No evidence: no relevant study of acceptable scientific quality available.

Because of a dearth of theoretical data in the primary empirical studies, it proved possible to use this rating scale only in the descriptive phase of the review.

### Realist analysis

In order to identify aspects of each empirical study that were key to the intervention’s success (or which explained its failure or partial success), we initially planned to extract data on (1) the nature of the intervention; (2) aspects of the context in which it was implemented; and (3) how the intervention was assumed (or shown) to work. Ideally, such data would be found prospectively in the methods and findings sections (in other words, both data collection and analysis would have been theory-driven). However, when this was not the case, we used the more speculative source of the retrospective explanations offered by authors in the discussion sections of their papers. In this way, we developed a preliminary set of ‘candidate theories’, for which we then sought affirming or refuting evidence from further studies.

With a view to identifying data that would help us assess the plausibility and strength of evidence for particular context–mechanism–outcome configurations, we developed and refined a data analysis matrix, initially oriented to the candidate theories identified above and adding further theories as these emerged. When developing and populating this matrix, FM and TG held regular discussions and considered whether key data items pertained to context, mechanisms or outcome.

Ideally, a realist analysis would surface a number of candidate theories from empirical studies and then find additional studies that had prospectively tested the same theories. The analysis would then focus on which of the initial candidate theories was supported by empirical evidence; some theories would be rejected and others extended and refined, producing a definitive list of programme theories. In the study reported here, the empirical evidence base was insufficient to support systematic testing of candidate theories, so these theories were presented as emerging hypotheses (rather than as definitive statements) about what works for whom in what circumstances.

Since very few primary studies had prospectively identified and tested theories, tentative theory was built from the descriptive data by considering the ‘barriers’ and ‘facilitators’ identified in the results sections of primary studies along with authors’ reflections on the how and why these had played out (usually found in the discussion sections of those studies). We also considered relevant policies and how these had unfolded, to identify the ‘theories incarnate’ embedded in policy programmes. Finally, as tentative theories emerged, we returned to our sample of primary studies and looked for empirical data to support them and also disconfirming data that might refute (or require us to modify) them. An example of how this was done is shown in Box 1.

## Box 1: Example of how we built tentative theory from primary studies

Most studies of pharmacist behaviour consisted of interviews with pharmacists asking whether and how they provided smoking cessation support or other extended role activities, what the pharmacists thought facilitated or interfered with this behaviour, and a commentary on these findings.

One review, for example, described primary interview studies in which pharmacists had talked confidently about their dispensing role but expressed reservations about their extended role. The latter uncertainties, suggested the reviewers, “*…were related to pharmacists’ undergraduate training, which was primarily based on the biomedical model of health* […and other influences].” [13].

To explore the contextual influence of undergraduate training further, we returned to our sample of papers and looked for any mention of this influence. A narrative review undertaken as background to an empirical study published 11 years later summarised how the same theme had emerged as significant in further studies [3]. In sum, and consistently across studies, when asked to account for their reluctance to take on a public health role, pharmacists emphasised that they had been trained as dispensers of medicines and found that this jarred with the health promotion role they were now expected to adopt.

We also identified from before-and-after studies of pharmacist training two key attitudinal changes that were consistently improved with training, namely ‘gives broader (less biomedical) definition of health’ and ‘views own role as more patient-oriented (as opposed to product-oriented)’. These findings supported the emerging theory that training influences the kind of pharmacist the person feels they are.

The Saramunee paper also highlighted the Modernising Pharmacy Careers initiative in England (which began in 2009), a major component of which was shifting the focus of education beyond a biomedical model in order to prepare pharmacists for the extended role expected of them by policymakers [14]. In other words, there was already a ‘theory incarnate’ in pharmacist training policy. This had not been empirically tested but reflected the professional wisdom of pharmacy educators.

Finally, we prospectively looked for disconfirming data (that is, data that would challenge the theory that a ‘biomedical’ undergraduate education helps create a more ‘biomedical’ pharmacist identity) in every study in our sample, and found none.

## Results

### Nature of dataset

Our final sample consisted of 66 papers describing 74 studies. These consisted of the following (some papers described more than one study type):

- 12 systematic reviews (5 of RCTs of pharmacist-led behavioural interventions for smoking [15–19], 1 of process elements of such interventions [20], 3 of pharmacist-led behavioural interventions other than smoking cessation [15, 19, 21], 1 of the scope of pharmacy practice [4], 1 of pharmacists’ perceptions [13], and 1 of qualitative studies of the patient experience [22]);
- 6 reviews not described as ‘systematic’, of pharmacy business models, pharmacist scope of practice, pharmacist training programmes or the process elements of RCTs [2, 23–27];
- 18 RCTs, of which 14 related to smoking cessation [28–41] and 4 to other behavioural interventions [42–45];
- 1 cost-effectiveness study linked to a RCT [40];
- 1 paper describing additional process detail on a RCT [46];
- 12 evaluations of pharmacist training courses, using either pre-post classroom assessments or ‘mystery shopper’ assessments of performance in practice, comprising 2 linked to RCTs [28, 29] and 10 before and after studies [47–56];
- 7 papers reporting quantitative surveys (6 of pharmacists [57–62] and 4 of service users [57, 58, 63, 64]);
- 6 papers describing qualitative studies, 5 based on semi-structured interviews (3 of pharmacists [3, 65, 66], 1 of pharmacy owners [67], 2 of service users [3, 65], 1 of researchers [68]) and 1 a focus group study of service users [69];
- 2 in-depth case studies [70, 71];
- 2 business models [67, 72];
- 1 paper describing the development of a complex intervention [73].

We classified the main underpinning academic discipline of the 65 papers as pharmacy practice (41), public health (7), psychology (5), medicine (including respiratory medicine and general practice (4), business and management (5), health policy (2), and health economics (2). In sum, most of the papers in this dataset described studies that had been undertaken by pharmacists and published from schools of pharmacy, and which were primarily concerned with demonstrating what pharmacists and their staff could do. The included primary research studies covered work in 10 countries, including UK (18), USA (15), Australia (5), Canada (4), and Ireland, Japan, Netherlands, Qatar, Sweden, Peru and Thailand (1 each). Systematic reviews and commentaries covered additional countries. One international comparative study covered 27 European countries [72] and a ‘grey literature’ report from the International Pharmaceutical Association covered advanced practice in 48 countries [25].

Of the 66 papers, 26 were classed as one star (significant flaws or so small and parochial that generalisability was in serious doubt). However, 21 studies were rated 2 stars and 19 (including 12 systematic and narrative reviews) were rated 3 stars. In sum, whilst many primary studies were unreliable, there was also a substantial body of adequately powered, well-conducted research on some though not all aspects of our research question, including several systematic reviews.

The sample of studies identified for this review was highly skewed towards the study of individual factors influencing behaviour (of either pharmacists or service users) and allowed us to draw definitive conclusions about these aspects of the review. However, the primary studies in our sample included very little empirical data on organisational and system factors. For example, surveys of attitudes, knowledge and perceptions were common, whereas no study had used organisational ethnography to study the complexities of the work environment or formally analysed the implications of proposed changes for pharmacy workforce planning (though some review articles and commentaries had speculated on these themes). In short, we had much data on ‘mechanisms’ but very little on ‘contexts’.

Few studies in our sample included theorisation by the authors (or contained sufficient descriptive detail to allow us to theorise) about the interaction between context, mechanism and outcome. Studies typically included only brief speculation about why an intervention might work better in some circumstances than others, and these discussions were invariably framed in terms of ‘barriers’ and ‘facilitators’. So, for example, ‘lack of time’ was widely considered to be a barrier to pharmacist-led smoking cessation provision, but authors did not go on to consider the circumstances in which lack of time might come to be less of a barrier. Thus, the literature provided useful information on the individual factors influencing pharmacist provision of this service, but not on how these factors interacted with one another.

For all these reasons, much of our synthesis is descriptive rather than theoretical and the realist analysis that follows is very preliminary.

### Descriptive findings

None of the studies included in this review had attempted a realist analysis. We felt that, in order to be faithful to what authors actually did, our findings should first be presented in terms of the research questions asked by the original authors (rather than in terms of the questions we ourselves subsequently imposed on their work). In this section, we describe the main research questions asked by other scholars in this field and judge the nature and quality of empirical findings.

#### Question 1: What is the impact on pharmacists of training them in smoking cessation support?

In the absence of specific training, pharmacists may be under-confident in their own ability to deliver smoking cessation support and may not consider it part of their role. Training has been shown to be positively received by pharmacists (strong direct evidence [54, 74]); it increases their knowledge (strong direct evidence: [49, 53, 54, 74, 75]), self-efficacy or confidence (strong direct evidence: [53, 54, 74, 75]) and intention to provide such support to their own clients (strong direct evidence: [53, 55]). These changes appear to be sustained over the medium term (moderate direct evidence [53, 56]). A well-designed training course may shift the pharmacist from a biomedical and product-oriented perspective – that is, considering their own role in terms of the dispensing of drugs and devices – to a more public health and patient-oriented one – that is, considering their role in terms of promoting health in the local community and supporting individuals with lifestyle change (moderate direct evidence: [13, 53]).

Pharmacists trained in smoking cessation support are more likely to deliver it, spend longer on it and adhere to evidence-based standards than those who receive no training (strong direct evidence: [47, 49, 52, 53, 55]).

It is noteworthy that in one study the number of pharmacy users actually counselled per pharmacist trained was very low (limited evidence [52]). A systematic review covering smoking cessation training and other behavioural interventions by pharmacists noted the near-absence of evidence on what is actually delivered to pharmacy customers following a training course (moderate direct and strong indirect evidence [74]).

#### Question 2: What is the perspective of patients and the public on pharmacy-based smoking cessation support and other non-dispensing roles of the pharmacist?

Members of the public who are counselled by a trained pharmacist about smoking cessation are likely to value the intervention, to find it helpful and to consider it appropriate that the pharmacist delivered it (limited evidence [55]). The same is true of other behavioural counselling – for example in support for asthma self-care (limited evidence [64]). Despite this, some pharmacy-based quit programmes are not widely used (strong direct evidence [52, 57, 63, 65]). Surveys of pharmacy users suggest that they may be unaware of the extended services provided by pharmacists (moderate direct evidence [3, 57, 69]); some lack confidence in the pharmacist’s non-dispending role and may even view the pharmacist as someone with limited skills, who works – or ought to work – under a GP’s instructions (moderate direct evidence [3, 65, 69]). The public’s top priority for pharmacy services is reliable supply of prescription medicines (strong direct evidence [3, 57, 58, 64, 65, 69]); and they have concerns about the level of privacy in pharmacy outlets – which may not be fully justified, as many are unaware that pharmacists have rooms for consulting in privacy (moderate direct evidence [3, 65, 69]).

#### Question 3: What do pharmacists perceive are the barriers to delivering smoking cessation support?

Surveys of pharmacists have identified a number of perceived barriers to delivering smoking cessation support as part of business as usual in their high-street pharmacies. These include personal characteristics of the pharmacist, namely a perceived need for (further) training (strong direct evidence [3, 13, 59, 61]); perceived lack of interest from patients (strong direct evidence [59, 60]), perception that behavioural counselling is beyond their role (strong direct evidence [3, 13, 61]), unwillingness to put a strain on their relationships with ‘customers’ (moderate direct evidence [27, 65]), and a dislike of counselling (limited evidence [61]). They also include perceived features of the pharmacy and its local environment: lack of time (strong direct evidence [3, 13, 59–61]), lack of space – especially for consulting in private (strong direct evidence [3, 13, 61]), difficulty in identifying which customers are smokers at point of care (strong direct evidence [59, 61]), lack of support staff (limited evidence [61]), lack of specific remuneration (strong direct evidence [13, 27, 59]), other more pressing pharmacy duties, especially the high workload of dispensing (strong direct evidence [3, 27, 61, 65]), lack of ongoing relationships with customers (strong direct evidence [13, 61]), lack of opportunity to network with other primary care professionals (moderate direct evidence [3, 13]), lack of support from the manager or owner of the pharmacy (limited evidence [61]), excessive patient demand (limited evidence [65]), lack of support from a local health promotion unit (limited evidence [13]), and lack of national and/or local publicity on what extended services were available at pharmacies (limited evidence [3]).

One study found that whilst most pharmacists have a room for consulting in private, this was considered ill-suited for social interaction and in practice was typically used as a place to escape from the stressful environment of the busy pharmacy, rather than for patient interaction (limited evidence [65]). The education of undergraduate pharmacists was also identified as a contextual barrier: respondents in one study viewed their undergraduate education as narrowly biomedical and drug-focused, making it difficult for them to develop a public health perspective on their professional role (moderate direct evidence [13]).

#### Question 4: What do pharmacists believe would help them to deliver smoking cessation support?

In surveys, pharmacists have identified a number of ‘facilitators’ that would help them deliver smoking cessation counselling. These include characteristics of the pharmacist: a personal identity that embraces the public health role (moderate direct evidence [13]), patient-centred values (moderate direct evidence [13, 66]) and a strong sense of professional ethics (limited indirect evidence [66]); a perception that smoking cessation is ‘medicines-related’ and hence part of the professional jurisdiction of the pharmacist (moderate direct evidence [13]), a positive attitude to smoking cessation counselling (strong direct evidence [13, 18, 61]), a belief that the counselling will be effective (strong direct evidence [18, 61]) and speaking the same ethnic language as the target customer(s) (limited evidence [13]). They also include characteristics of the pharmacy: low job stress (moderate direct evidence [13]), the extent to which the pharmacist can work with known, regular customers (moderate direct evidence [13]), and the introduction of new pharmacy roles, especially the accredited pharmacy checking technician to help with the ever-increasing workload of dispensing (limited evidence [3]). Pharmacists have acknowledged the worsening ethical dilemmas in their role as a result of escalating commercial pressures, contractual changes and public demand (moderate indirect evidence [3, 65, 66]).

Whilst one study identified a ‘community’ ethos and culture, namely, small size, stable local population and tradition of providing preventive services (moderate direct evidence [13]), as supportive of a public health role of the pharmacist, respondents in another study considered that large pharmacy chains were better placed to develop the standard operating procedures, promotional material and training packages to support the delivery of novel services (limited evidence [3]). System factors perceived by pharmacists to facilitate their provision of smoking cessation counselling include endorsement of the extended role of the pharmacist by national directives and/or a professional body (moderate direct evidence [13]) and the nature and quality of the local primary care network (limited evidence [3]), since “*collaboration is important for signposting or for referring into existing systems, ensuring holistic care, avoiding duplication, ensuring targeting of different populations, and enabling individuals to select their preferred public health service provider*” ([3], p. 279).

#### Question 5: How do organisational and system factors influence uptake and delivery of smoking cessation counselling by pharmacists?

We found little empirical evidence on this question but we did identify several reviews and commentaries considering policy drivers and business models.

In the UK, for example, there have been four policy drivers towards an extended role of the community pharmacist (towards either specific services such as smoking cessation support or, as envisaged by some, a comprehensive population-wide public health role including health promotion, disease prevention and disease management [26]) in recent years: the 2005 NHS Pharmacy Contract; the notion of a ‘responsible pharmacist’ who is present on site and supervising dispensing; the introduction of Pharmacists with Special Interest; and the regulation of the pharmacy workforce including separate licensure of pharmacy technicians (limited evidence [2]). Similar but not identical shifts in the scope of pharmacy practice are occurring in other western countries (moderate direct evidence [67, 72]).

The 2005 contract in the UK represents a number of significant shifts for community pharmacies as businesses: in the source of pharmacists’ income (from margins on dispensed medicines to fees for services delivered); in the definition of the pharmacist’s role (from ‘dispenser of medicines’ to ‘prescriber’, ‘supporter of self-care’ and ‘educator’); in the nature and extent of clinical governance requirements (a significant increase in the amount of paperwork for accountability, quality assurance and accreditation), requiring organisational knowledge and standard operating procedures; in skill mix (especially in relation to assistants and technicians, as well as increasing diversification and specialisation); and in the requirement for training. These changes occur in the context of a rapid shift in recent years from single, owner-occupied pharmacy practices to larger chains – which now dominate the UK sector and which, as noted above, may be better able to weather these extensive changes in the business context.

In turbulent times, the ability to adapt rapidly and appropriately to external policy changes is key to the survival of a pharmacy as a business. A qualitative interview study from Australia sought to identify the capacity of current pharmacy business models, and in particular the dimensions of organisational flexibility within these models, to integrate both the traditional product (medicines) focus and the new extended role (services) of pharmacists (limited evidence [67]). The authors identified four kinds of organisational flexibility from the business and management literature:

- Steady-state (limited flexibility, centred on the status quo, resistant to any change);
- Operational flexibility (able to react to short-term market demands but incapable of making significant structural or strategic changes);
- Structural flexibility (able to use managerial capabilities to alter a firm’s structure to respond to internal and external pressures, hence can embrace medium-term change);
- Strategic flexibility (able to engage proactively and strategically to accommodate change and embrace opportunities, hence can embrace long-term change).

The authors found four types of community pharmacy in their (Australian) setting: the traditional, small dispensing pharmacy that offers few additional services and representing the steady-state; larger retail pharmacies with higher throughput of goods and typically exhibiting operational flexibility to increase and vary the product range in retail sales; the ‘healthcare solution pharmacy’ (based on creating a professional image as healthcare providers and the provision of services, and perhaps offering niche services from specialist employees); and the networked pharmacy (two or three pharmacies connected through a shared ownership structure) – different pharmacies offer different products and services based on reaching critical mass (or economies of scale) and position themselves effectively to the local competition. Both healthcare solution pharmacies and networked pharmacies tend to exhibit either structural flexibility (i.e. they develop services in a few key areas and change structure to implement these) or strategic flexibility (i.e. they pro-actively plan and integrate new services into the brand image of the pharmacy and support these through development of internal systems, staff and practices). Strategic questions include skill mix, the structural and material environment of the pharmacy, return on investment, and marketing and communication activity. Importantly, the emergence and adoption of new business models in existing and new pharmacies in the local environment stimulates a shift in the business model of neighbouring pharmacies since competition promotes service development (moderate direct evidence [67]).

Whilst business models in the UK and elsewhere for delivering pharmacy products and services have changed rapidly since the early 2000s, there is some evidence that models of financial remuneration have lagged behind these changes. A systematic review of remuneration models for pharmaceutical professional services in different countries found that, in most existing models, pharmacists’ income remains linked primarily to the medicines dispensed, though in many countries there is now a significant income stream linked to managing the use of these medicines and liaising with general practice to maximise safety and minimise waste (strong direct evidence [76]). Most countries regulate remuneration for services only when the medicine is paid for under a national reimbursement scheme. Two reasons for the absence of attractive remuneration schemes for pharmacy professional services (defined as “*the contribution of pharmacists and their assistants to medicines therapy as a part of the total care supplied to patients, in co-operation with physicians and other health care professionals, with a view to optimizing the efficiency, the effectiveness and the safety of medicines*” [76]) in many countries are a fixed overall budget (hence, increased funding for professional services will reduce the amount available for dispensing and medicines management) and lack of evidence on the cost-effectiveness of these services (strong direct evidence [76]).

The discussion and policy papers in our sample highlight an inherent ambiguity in the pharmacist’s role, which combines retail (hence, the business incentive to offer products of limited therapeutic value to meet consumer demand, and to foster a ‘customer is always right’ ethos) with professional services (hence, the pressure to be ‘evidence-based’ and challenge consumer choices where appropriate) [3, 27]. This role ambiguity is perceived to be exacerbated by imposed targets and a task-oriented approach to policy [76].

The issue of professional jurisdiction was a key theme in the discussion papers in our sample. When one profession seeks to take over an aspect of the core practice of another profession, the latter may view this as competition. General practitioners in the UK have expressed confidence in pharmacists’ core role of dispensing and monitoring medicines but voiced concerns about their competence to provide pharmacy professional services (limited evidence [3]). In some countries, notably the USA, competition has been formally managed by the introduction of Collaborative Practice Agreements between physicians and pharmacists, though such agreements are not yet widespread (strong direct evidence [25]). The introduction and extension of professional recognition, credentialing and privileging may also aid the development of an accepted professional jurisdiction in pharmacy professional services, though progress on this matter is slowed by poor agreement across countries in the definition of pharmacy professional services and the nature and level of training required to deliver it (strong direct evidence [25]).

### Realist analysis: what might work for whom?

Based on the above descriptive synthesis, we identified five mechanisms by which a programme to deliver smoking cessation support via community pharmacists might achieve its goals (typically measured in terms of clients counselled, quit rates achieved and quit rates sustained) and built tentative theories to explain the influence of context on these mechanisms (see example in Box 1). The mechanisms were pharmacist identity, pharmacist motivation, pharmacist capability (knowledge, competence), clinician confidence and public trust. Whilst all these mechanisms were identified primarily from the empirical studies included in this review, they also align with a wider literature – which we were not resourced to review comprehensively – on the psychology of behaviour change and the adoption of innovations (discussed in relevant sections below), and on policy for pharmacist education and development [77]. We consider the five mechanisms in turn below.

#### Mechanism 1: Pharmacist identity

Most studies in our sample studied pharmacists rather than other staff employed in pharmacies (this is probably due to the relatively recent introduction of the role of pharmacy assistant). Pharmacists are more likely to want to deliver smoking cessation if they see themselves as having a public health function (and not merely as a dispenser of medicines); if they are patient-oriented rather than product-oriented and if they have a strong sense of professional ethics. The primary studies in our sample identified three key contextual influences on these elements of identity. First, undergraduate education that is oriented towards nurturing these characteristics will tend to produce new pharmacy graduates who are likely to accept their extended role and gain fulfilment from it. Second, this sense of identity is likely to be sustained if professional bodies embrace the extended role and engage proactively in the postgraduate development of pharmacists in a way that explicitly goes beyond dispensing. Thirdly, it is also likely to be sustained if policymakers view pharmacists as professionals (and the delivery of public health services as a professional role) rather than viewing pharmacists as individuals who could be incentivised to perform a technical task, and if they engage in dialogue with professional bodies to address conflicts between pharmacists’ professional role and their business role.

#### Mechanism 2: Pharmacist capability

Pharmacists (or staff employed by them) are more likely to want to deliver smoking cessation services, and to actually deliver them, if they are knowledgeable about the topic, confident in their own ability to deliver behavioural counselling (self-efficacy) and have good communication skills, and if they believe their intervention will be effective. Contextual influences that are likely to affect these elements of pharmacist capability relate to the quality, depth and breadth of the training at both undergraduate and postgraduate level, whether such training is accessible (and actually accessed) throughout the professional life of the pharmacist and the extent to which it addresses skills and attitudes as well as just knowledge.

#### Mechanism 3: Pharmacist motivation

The empirical evidence shows that motivated staff are more likely to deliver smoking cessation counselling. This motivation can be divided into professional motivation, personal motivation and business motivation. Contextual factors likely to enhance professional motivation include the involvement of other pharmacists (and pharmacies) in smoking cessation, setting a strong professional norm against which other pharmacists would benchmark their own practice, and receiving positive feedback at professional appraisals. Personal motivation is likely to be higher in contexts where the trade-off between remuneration and the paperwork of claiming this remuneration is favourable. Factors likely to enhance business motivation include a business model that makes it worthwhile to invest in space, staff training and necessary infrastructure.

It should be noted that most studies in our sample were undertaken on pharmacists at a time when the predominant business model (in the UK and elsewhere) was the small ‘owner-occupied’ dispensing pharmacy. Motivation of pharmacists in this position may be different from that of a salaried pharmacist in a large chain or a pharmacy assistant.

The above three mechanisms, all relating to pharmacist behaviour, resonate with the wider theoretical literature on learning and behaviour change. In the theory of planned behaviour, Ajzen proposed that, if people evaluate a behaviour as positive in terms of outcomes and importance of that outcome (attitude) and if they think significant others whom they value want them to perform the behaviour (subjective norm), this results in a higher intention (motivation) and they are more likely to do so, provided they perceive they have control to do so and that any external constraints to achieving the suggested behaviour are removed [78]. Bandura’s social learning theory proposes that people are more likely to change their behaviour if the training addresses expectancies about the likely benefits of the intervention and includes (in addition to factual knowledge) exposure to positive role models, attention to particular skills, development of self-efficacy (confidence in one’s ability to apply those skills), and environmental support to use those skills [79]. Rogers’ diffusion of innovations theory further expanded the complex and powerful phenomenon of peer influence among health professionals [80].

Also relevant is Grol’s five-stage theory of change among health professionals, comprising orientation (in which the potential adopter becomes aware of, and interested in, a new approach), insight (in which they come to understand the innovation and also understand the limitations of their current practice), acceptance (in which they develop a positive attitude to change as well as the intention to change), change (in which they start exhibiting the new behaviour and confirm its value), and maintenance (in which they work to embed the new practice into personal and organisational routines) [81]. Whilst none of the primary studies we reviewed explicitly set out to test these theories of behaviour change, their findings are very consistent with this wider theoretical literature.

#### Mechanism 4: Clinician confidence

As noted above, smoking cessation interventions delivered in community pharmacies are critically dependent on a strong referral pathway from general practitioners [3], but empirical evidence suggests that this pathway, even in a research setting, is often weak [52, 57, 63, 65]. In one study, this was explained by the fact that referring general practitioners had low confidence in pharmacists’ capability to deliver smoking cessation support [3]. This may in turn partially reflect pharmacists’ own lack of confidence and/or willingness to take on a smoking cessation role (described above). The primary studies in our sample suggest that clinician confidence may be enhanced by positive messages in professional journals about the pharmacist’s extended role; a positive attitude and actions from doctors’ professional bodies; and a local primary care network that understands, acknowledges and is willing to signpost and affirm this role among community pharmacists.

#### Mechanism 5: Public trust

As noted in the descriptive findings, primary studies suggested that public confidence and trust in pharmacists to deliver smoking cessation support is limited. Much of that primary data emphasised the public’s lack of awareness and exposure to public health and health promotion activity by pharmacists. A focus group study in the UK explored reasons for lack of trust in pharmacists relative to general practitioners [69]. It identified numerous system-based factors that reinforce patient trust and confidence in general practitioners, including the registered list system, appointment systems, the general practice expert and gatekeeper role, the physical environment of the practice, and good understanding of the training of doctors. These factors all helped to foster familiarity with a specific doctor or practice, which allowed interpersonal trust to develop. In contrast, participants had much more limited and less consistent exposure to community pharmacists and more limited knowledge of how they were trained and their systems for practice. Other studies in our sample suggested additional contextual factors that will tend to engender public confidence and trust, include clear, consistent and positive messages in the media about the pharmacist’s extended role and positive messages on this topic from general practitioners.

**Fig. 2:**
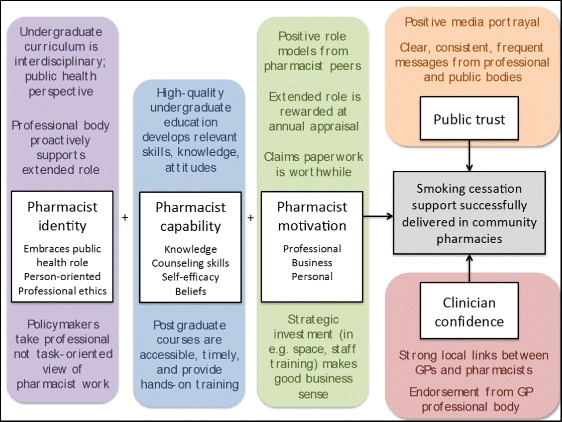
Unifying model showing key influences on successful delivery of smoking cessation support in community pharmacies. *White boxes* represent mechanisms; *coloured boxed* represent contextual influences that make each mechanism more likely to have effect; the *grey box* is the outcome

Figure 2 offers a provisional unifying model of the above five mechanisms and the contextual influences on them. For high-quality smoking cessation support to be delivered to significant numbers of people in a community pharmacy setting, three things need to happen. First, pharmacists need to be willing and able to engage in this activity – a function of their professional identity, their specific capability to perform the role and their motivation (which will be influenced both by professional norms and by the practical and financial reality of delivering on this aspect of their role in a busy pharmacy setting). Second, there needs to be a clear and well-used referral pathway, especially from local general practices; this will depend on clinicians’ confidence in their pharmacist colleagues and will be enhanced by positive messages from their professional bodies and by increased interprofessional interaction. Finally, the public needs to have high confidence and trust in the extended role of the pharmacist – something that may currently be lacking but which could be increased by systematic attention to raising awareness and promoting positive media messages.

## Discussion

### Summary of findings and comparison with previous findings

This qualitative review, which links to the findings of a parallel quantitative review for the Cochrane EPOC group, included a wide range of study designs written up in 66 papers. It summarises and extends a substantial existing literature of systematic reviews (based on dozens of primary studies) on community pharmacy-based smoking cessation services. Most empirical studies on pharmacist involvement in smoking cessation were small trials undertaken by pharmacists; they focused on the impact of training on the confidence and capability of pharmacists and their staff (improved); pharmacists’ attitudes to their extended role (mixed); pharmacists’ perceived barriers to delivering smoking cessation (multiple, including insufficient training, insufficient time or space, insufficient interest from patients, inadequate remuneration and more pressing priorities), and pharmacists’ views on what would help them deliver such a service (multiple, including training, professional body support, more time and space, and additional staff roles). There was very limited empirical evidence on organisational and system influences on this extended role.

Our findings substantiate some key recommendations of previous systematic reviews: that pharmacists and their staff, when properly trained and supported, are capable of delivering the non-dispensing elements of a smoking cessation service as well as providing nicotine replacement products [4, 15–18, 20, 74, 75, 76]; that training improves their confidence and performance in this area [74, 75]; that there may be role ambiguity and/or issues of professional identity when pharmacists are invited to take on non-dispensing roles [13]; that primary studies have, to date, produced a very limited evidence base on the real-world implementation of smoking cessation services (for example, the active components of interventions have been poorly described and rarely theorised) [4, 13, 17, 20, 74]; that pharmacies may be run according to different business models but the evidence base on how these different models support non-dispensing pharmacy services is limited [4, 76]; and that there are policy implications of a major change in the professional jurisdiction of pharmacists [4, 17].

Our review adds to the existing literature by going beyond the search for ‘active components’ of a generic smoking cessation intervention and offering a more nuanced and theoretically informed analysis in a provisional realist analysis. Key findings from our realist analysis are that, firstly, for the community pharmacy to become the site of smoking cessation support, the pharmacist needs to view themselves as a public health professional rather than (merely) a dispenser of medicines – something that is far more likely to happen if undergraduate training, professional bodies and national policy depicts them in this way and endorses the role positively. Secondly, that training (oriented to increasing knowledge, communication, belief in one’s ability to deliver the intervention and belief in the efficacy of the intervention) is more likely to be effective at both undergraduate and postgraduate level if courses are affordable and accessible and if a broad curriculum is provided that goes beyond ‘tasks and facts’. Thirdly, that pharmacists are more likely to be motivated (from a professional, business and personal perspective) to deliver smoking cessation services if other pharmacists locally and nationally are also doing so; if the work is received positively at appraisal and performance review; if structural and logistical issues (time, space, priorities) are addressed; and if the work is adequately remunerated and avoids excessive claims paperwork. Finally, the shift to include smoking cessation services – as with other aspects of the non-dispensing role of the pharmacist – must have the confidence of other health professionals (especially general practitioners) and the trust of the wider public; this is more likely to happen if clear, consistent and positive messages are provided from the wider community of local health providers, doctors’ professional bodies and the media.

Early qualitative work by our own group on the STOP study confirms a number of barriers identified in this realist review, including, for example, pharmacy workers’ reluctance to risk threatening the relationship with ‘customers’; difficulties experienced by (often junior) pharmacy staff identifying who is a smoker at the pharmacy counter; the crucial importance of support from top and middle managers; and low public awareness of, and confidence in, the community pharmacy as a place to access smoking cessation support [82]. Our findings also confirm that a strong sense of professional ethics and a ‘public health’ orientation are associated with keenness to provide smoking cessation services; and that in multi-ethnic areas, pharmacy assistants (who may deliver the smoking cessation intervention) are more likely than pharmacists to reflect the diverse ethnic backgrounds of customers [82].

### Strengths, limitations and future research directions

This review followed the international RAMESES guidelines for realist synthesis [9]. The strengths of realist review have been described in detail in that paper. Briefly, such reviews are highly nuanced and go beyond the grand mean of ‘effect size’ to consider what interventions will work well (and less well) for whom in that circumstances – hence they can inform how interventions might be optimised for local delivery and impact maximised. This review included a wide range of study designs undertaken by different disciplinary teams with different goals, and has begun to tease out key interactions between the theoretical mechanisms of success and the context in which the intervention could or might be delivered. As such, it makes an important potential contribution to informing the development of policy both in the UK and elsewhere.

The main limitation of this review is that the primary data were largely atheoretical and highly skewed towards the assessment of the attitudes, capabilities and response to training of individual pharmacists. Most studies included little or no contextual detail with which to build theory about organisational and system influences (for example, only two of 61 primary studies mention the word ‘contract’ [2, 35] and very few systematically addressed the business aspects of pharmacist-led smoking cessation services. As noted in the methods section, naturalistic studies of how pharmacy work is conducted in practice were absent from our sample. These limitations mean that the context–mechanism–outcome links proposed in our findings section are preliminary and should be subject to further empirical testing.

Potentially, a wider range of literature (both theoretical and empirical) could have been brought to bear on the development of our theoretical model, since realist reviews may include any studies that contribute to the development of theory on a topic, whether or not they themselves cover the same topic. Whilst we did include studies of pharmacy practice beyond smoking cessation support where these contributed to theory building, we were not resourced to extend the review further.

This review was undertaken in parallel with the iterative development and refinement of a complex intervention for the STOP trial. Early interviews in preparation for the STOP trial have confirmed the findings of previous studies in relation to pharmacists’ (and pharmacy workers’) perceived need for additional training and support if they were to deliver an effective smoking cessation service [82].

The findings from this review that identity, capability and motivation of the pharmacist were key mechanisms influencing the delivery of smoking cessation interventions, and that these were mediated by the type, frequency and duration of training as well as by numerous contextual influences, contributed to the design of the pharmacist training intervention. These constructs have been integrated within the intervention being delivered in the ongoing STOP trial (which was guided by both the ‘capability, opportunity, motivation and behavior’ model of behaviour, incorporating key domains of capability, opportunity and motivation, as well as more detailed behavioural theories such as social cognitive theory and self-determination theory [83]). The intervention is described in detail in a separate publication (in preparation).

## Conclusion and recommendations

Smoking cessation support in community pharmacies has been extensively studied but few firm conclusions are yet possible on how best (if at all) to introduce this as a national policy. There is now an extensive and reasonably robust existing literature on individual behaviour change (how to train and support pharmacists and their staff, and the impact on patients), and also on individual pharmacists’ attitudes and perceived barriers to change.

It is evident from our provisional model that numerous factors will need to combine and interact over time to generate a successful programme of high-quality, pharmacist-led smoking cessation support. These include (but may not be limited to) policy support, undergraduate and postgraduate training of pharmacists, professional sharing of practice and growing stakeholder confidence. A number of ongoing natural experiments have the potential to generate case study data on how these influences play out in different national and professional settings – and hence inform refinement of the provisional model proposed here.

A reviewer of an earlier draft of this paper pointed out the similarities between smoking cessation support and alcohol advice in a pharmacy setting. In both cases, the pharmacist is required to take on additional tasks and also a person-focused and public health perspective. One way of doing this in a time-efficient manner might be to include questions about smoking and alcohol in pharmacist-led medication reviews. Such an approach has not (to our knowledge) been tested specifically, though there is wider evidence that a ‘care bundle’ (that is, a small set of evidence-based interventions for a defined patient population and care setting) can greatly improve both processes and outcomes of care [84].

Our findings suggest that a high priority for further research is the organisational and system context for community pharmacy-based smoking cessation (especially how different business models for delivering pharmacy services may shape, enable and constrain pharmacists’ extended role) and on the costs and cost-effectiveness of a pharmacist-led model compared to other modes of delivery. Finally, a striking finding in the sample of primary studies identified for this review was the near-absence of theoretically informed studies; further empirical studies of pharmacist-led smoking cessation should see both to apply and test relevant theories of individual and organisational change. The ongoing STOP trial is addressing organisational influences via a detailed process evaluation and also includes a cost-effectiveness analysis [5].
